# Can temperature explain the latitudinal gradient of ulcerative colitis? Cohort of Norway

**DOI:** 10.1186/1471-2458-13-530

**Published:** 2013-05-31

**Authors:** Geir Aamodt, May-Bente Bengtson, Morten H Vatn

**Affiliations:** 1 Department of Epidemiology, Norwegian Institute of Public Health, Oslo, Norway; 2 Department of Medicine, Tønsberg County Hospital, Tønsberg, Norway; 3 The Institute of Clinical Epidemiology and Molecular Biology (EpiGen), Campus Ahus, Institute of Clinical Medicine, University of Oslo, Oslo, Norway; 4 Medical Clinic, Oslo University Hospital, Rikshospitalet, Oslo, Norway

**Keywords:** Epidemiology, Colitis, Ulcerative colitis, Climate, Temperature, Precipitation, Altitude, Hygiene hypothesis

## Abstract

**Background:**

Incidence and prevalence of ulcerative colitis follow a north–south (latitudinal) gradient and increases northwards at the northern hemisphere or southwards at the southern hemisphere. The disease has increased during the last decades. The temporal trend has been explained by the hygiene hypothesis, but few parallel explanations exist for the spatial variability. Many factors are linked to latitude such as climate. Our purpose was to investigate the association between variables governing the climate and prospectively identified patients.

**Methods:**

In this study, we used a subset of the population-based Cohort of Norway (n = 80412) where 370 prevalent cases of ulcerative colitis were identified through self-reported medication. The meteorological and climatic variables temperature, precipitation, and altitude were recorded from weather stations of the Norwegian Meteorological Institute. Summer temperature was used to capture environmental temperature.

**Results:**

Summer temperature was significantly related to the prevalence of ulcerative colitis. For each one-degree increase in temperature the odds for ulcerative colitis decreased with about 9% (95% CI: 3%-15%). None of the other climatic factors were significantly associated to the risk of ulcerative colitis. Contextual variables did not change the association to the prevalence of ulcerative colitis.

**Conclusions:**

The present results show that the prevalence of ulcerative colitis is associated to summer temperature. Our speculation is that summer temperature works as an instrumental variable for the effect of microbial species richness on the development of ulcerative colitis. Environmental temperature is one of the main forces governing microbial species richness and the microbial composition of the commensal gut flora is known to be an important part in the process leading to ulcerative colitis.

## Background

Ulcerative colitis is characterised by a dysfunction of the intestinal epithelium barrier, resulting in chronic inflammation [1,2]. It is assumed that the disease is caused by an imbalance between the immune system and the commensal microbial flora in genetically susceptible individuals. Genome-wide association studies have identified several gene variants associated with inflammatory bowel disease including ulcerative colitis [3-5].

Several risk factors have been identified regarding ulcerative colitis. Among those are socioeconomic status [6], nutrition [7], and microbiology [8]. Recently, studies have shown associations with iron in the drinking water [9] and components as SO_2_ in air-pollution for development of ulcerative colitis in young people [10]. Smoking [11], breast feeding [12] and appendectomy [13] have shown to be protective factors, while perinatal infections [14] as well as gastroenteritis early in life [15] have been linked to increased risk for ulcerative colitis, all factors influencing the gut microbiota or the mucosal immune system. Risk factors and their influence on ulcerative colitis are listed in Table 1.

**Table 1:** List of demographic and environmental risk factors related to ulcerative colitis

**Variable**	**Comment**	**References**	**Present study**
Age, sex	More men than women, 25–35 years	Moum [56]	Included
Smoking	Protective (OR: 0.58)	Mahid [11]	Included
Appendectomy	Protective	Lopez [13]	Not included
Diet	Red meat, n-6 fatty acids, sweets increase risk n-3 fatty acids, fruits, vegetables decrease risk	Ng [58] (references therein)	Not included
Breast-feeding	Protecting	Klement [12]	Not included
Acute gastrointestinal infections	Risk factor. Campylobacter, Salmonella	Gradel [59]	Not included
Sun-exposure	Not investigated	-	Not included
Sosio-economy	Higher in urban, urban/rural change	Aamodt [19]	Included

See Ng et al. for review [58].

*OR* Odds ratio.

Country of residence and time period are important factors for the development of ulcerative colitis. Incidence rates are reported to be higher in northern Europe than in southern Europe [16] and a similar pattern has been demonstrated in the US [17]. Variability and clustering within countries have also been identified [18-21].

Temporal changes are even larger, reporting a 2–3 fold increase in the risk of disease from World War II up to now, in Olmsted County, Minnesota [22]. Among children with ulcerative colitis, rising incidence rates have been reported and also differences between countries [23].

In spite of obvious differences in incidence rates and latitude, few studies have focused on which factors that could explain the differences. Some have speculated that sun exposure or ultraviolet exposure and production of vitamin D could be potential factors, but these hypotheses are not tested properly [24].

Temporal trends in incidence rates have been explained by the “hygiene hypothesis”, stating that reduced exposure to microbial agents in childhood and consequently fewer infections, could lead to higher risk for the development of autoimmune diseases - like ulcerative colitis [25,26]. Relatively few studies have focused on exposure of hygiene-related risk factors such as infections in childhood, pets in childhood, family size, and vaccination and the development of ulcerative colitis, and no clear evidence is found [27-29]. Bernstein et al. reported significant associations between drinking unpasteurized milk and eating pork and risk of developing ulcerative colitis in univariate analyses, but these factors were probably confounded with factors which are not related to the hygiene [27]. Lopez-Serrano et al. observed that respiratory tract infections and gastroenteritis in childhood were protective factors [29].

Rook and Brunet suggested that a reduced exposure to normal microbes - “old friends” - corresponds to temporal changes in incidence rates for some autoimmune diseases [30]. The old-friends-hypothesis is also named the “extended hygiene hypothesis”. Both of these hypotheses are linked to species richness or biodiversity in our surroundings. Species richness, or biodiversity, is unevenly distributed on Earth, but still distributed according to some basic patterns such as the variability of environmental temperature, precipitation, area-size, and altitude [31].

The aim of the present study was to study the association between climatic factors and the prevalence of ulcerative colitis to improve our understanding of the geographic distribution of the disease in Norway. In a homogenous country like Norway, differences in diagnostic praxis and economic conditions are small, but the ranges of important climatic factors such as environmental temperature, precipitation, and altitude are substantial. Associations between these factors and risk of ulcerative colitis could therefore shed new light on the development of disease, and possibly support the extended hygiene hypotheses.

## Methods

### CONOR-database

**Figure 1:**
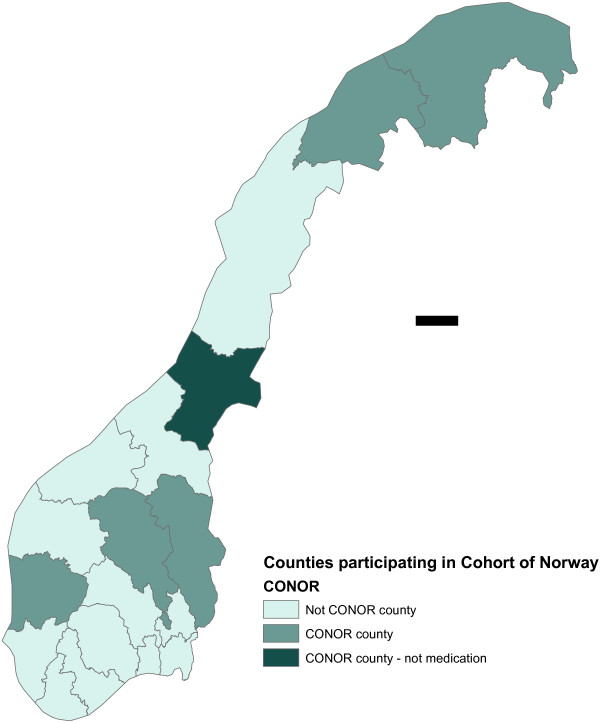
**Cohort of Norway counties.** The figure shows the different counties included in the Cohort of Norway collaboration. Seven out of 19 counties were included in ten health surveys. Self-reported medication was included in only eight of the ten health surveys.

The Cohort of Norway (CONOR) database was applied in this study. CONOR is a collection of ten different regional health studies conducted from 1994 to 2003 and from seven out of 19 counties in Norway (Figure 1). A total of 174 429 individuals participated in the studies, but only 80 412 individuals had complete data for the variables we were interested in. Physical examinations, including measurements of weight and height were conducted at screening. The participants also answered a set of common questions regarding their own health, self-reported diseases and diseases among family members, work, housing, types of occupation, medication, and reproductive history for women. Only eight of the health studies had information concerning medication. The CONOR database is described elsewhere [32,33].

### Identification of ulcerative colitis patients

We used the participants’ self-reported use of medication to identify prevalent cases of individuals with ulcerative colitis. As a part of the questionnaire, each participant could report a maximum of eight different medications they used on a regular basis. Individuals who reported that they used aminosalicylic acids were classified as prevalent cases. This medication was prescribed to most ulcerative colitis patients and Crohn patients with affected colon. We used the following ATC codes: A07EC01 (Sulfasalazine), A07EC02 (Mesalazine), A07EC03 (Olsalazine), and A07EC04 (Balzalazide). If a participant used one of these four medications he or she was classified as a clinical case of ulcerative colitis. The self-reported use of aminosalisylic acid was our dichotomous response variable.

### Other variables associated to ulcerative colitis

Smoking, education, gender, and age were included as potential confounders in the study. Smoking and education was part of the CONOR questionnaire. Smoking was categorized into two groups: ‘Daily smoker’ or ‘Never smoker’. Education was reported as number of years attending school. We have shown earlier that contextual variables, such as education, are associated to incidence of ulcerative colitis [19]. To accommodate the potential confounding effects of these contextual variables, we included the following variables for each municipality: percentages of inhabitants with more than 12 years education, mean income, urbanity, and urban/rural change. The urbanity variable is defined as the percentage of inhabitants in a municipality who live in a cluster of more than 200 people and where the distance between the houses is less than 50 meters (http://www.ssb.no). The urban/rural change variable is the relative change of urbanity for a municipality relative to the country, based on the national surveys in Norway from 1960 to 2001. Municipalities with positive values have experienced a faster pace of urbanization than the whole country during the actual period of time, while municipalities with values less than zero has experienced a slower pace of urbanization.

### Climate data

Climatic observations are reported in different ways, but we used figures based on measurements from 1960 to 1990. A total of 1683 weather stations by the Norwegian Meteorological Institute were included from the 441 different municipalities (eklima.met.no). We used the figures from the neighbouring weather station in two of the municipalities because they did not have their own weather stations. These two municipalities were small in size, located in a densely populated part of Norway, with short distance to the weather stations of the neighbouring municipality. We used the participants’ municipality as the geographic entity to link the climatic variables. The climatic variables for a given participant were thus the measurements reported from its municipality. For each municipality we computed an arithmetic average value based on the different measurement stations within each municipality.

The following variables were included in our analyses: yearly average precipitation, average summer temperature (July), and altitude. We used summer temperature because this variable has shown to be linked to species richness [31,34].

### Statistical analysis

We used standard chi-square-tests and t-tests to investigate if there were any differences in age, gender, education, and smoking habits between ulcerative colitis and non-ulcerative colitis participants. To model the risk of disease we used logistic regression models. To better accommodate the effect of area we fitted multi-level models including the municipalities as a random factor. To compare the different models we used Akaike's Information Criteria (AIC). To visualize the association between temperature and prevalence of ulcerative colitis, we used generalized additive models. We used R (2.15.0) for statistical analysis and ArcGIS (9.3) for visualization.

## Results

### Characteristic of the CONOR database

Table 2 shows summary statistics of the cases identified as ulcerative colitis and the controls. Only gender and smoking history were statistically different in the two groups. Ulcerative colitis was more frequent among men and among non-smokers.

**Table 2:** Summary of demographic statistics for the CONOR database stratified according to their ulcerative colitis (UC) status

		**UC**	**Non-UC**	**p-value**
Participants		370 (0.2%)	80152 (99.8%)	
Age	Mean (SD)	50.4 (14.0)	51.8 (12.9)	0.113
Gender	Males (%)	57%	50%	0.013
	Females (%)	43%	50%	
Education	Mean (SD)	12.0 (3.7)	12.4 (3.8)	0.094
Smoking	Daily smoker (%)	23%	29%	0.007
	Not smoker (%)	77%	71%	

The p-values in the last column result from statistical tests of equality between the two groups.

### Climatic factors

**Figure 2:**
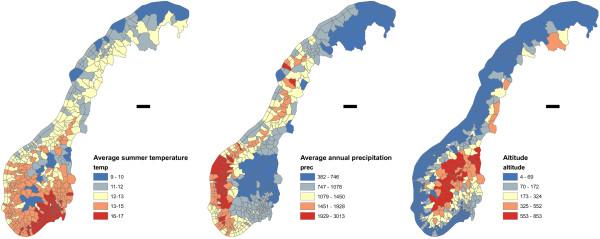
**Climatic variables in Norway.** The figure shows average values of summer temperature (C) and precipitation (mm) in Norwegian municipalities based on the 1960–90 observations at 1683 measuring stations in 441 municipalities in Norway as well as altitude (meters) for the different municipalities.

Maps of the climatic variables are shown in Figure 2. The figures depict average values of the three climatic variables with choropleth maps. For average summer temperature (July) the range was from 9.2 C to 15.5 C. Minimum and maximum precipitation was 382 and 2999 mm per year. Altitude varied from 5 to 832 meters above sea level.

### Ulcerative colitis and climate

**Figure 3:**
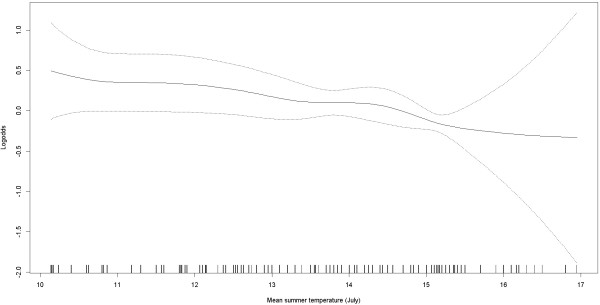
**Association between temperature and prevalence of ulcerative colitis.** The figure shows the association between mean summer temperatures based on registrations from 1960–90 and prevalence of ulcerative colitis based on Cohort of Norway. The y-axis shows the log-odds for prevalence of the disease.

The results from logistic regression models are shown in Table 3. For each of the climatic variables we included age, gender, smoking, and significant contextual variables in the model. Separate models were fitted for each climatic variables; summer temperature, annual precipitation, and altitude. Only summer temperature was significantly associated with the prevalence of ulcerative colitis. When summer temperature increases with one degree, the odds for developing ulcerative colitis decreases with 9%; OR = 0.91 (95% CI: 0.85-0.97). The set of contextual variables or including municipalities as a random variable did not change the effect measures between temperature and ulcerative colitis. The resulting AIC values were minimized for the simple model with summer temperature, smoking and gender as explanatory variables compared to multilevel models including contextual variables (data not shown). In Figure 3 we depict the association between summer temperature and (logit) prevalence of ulcerative colitis.

**Table 3:** Results from logistic regression analysis showing adjusted odds ratio of prevalence of ulcerative colitis dependent on different climatic variables

	**aOR**	**95% CI**	**p-value**
Summer temperature	0.91	(0.85–0.97)	0.014
Average annual precipitation (dm)	1.00	(0.98–1.02)	0.959
Altitude (per 100 m)	1.04	(0.95–1.14)	0.454

Separate models were fitted for each climatic variable, but adjusted for gender, smoking, and urban/rural change status. For each climatic variable we report adjusted odds ratios (aOR), 95% confidence intervals and p-values.

## Discussion

In this study we have shown that the prevalence of ulcerative colitis is dependent on summer temperature, but not the other climatic variables such as annual precipitation, or altitude. We have not found similar studies investigating the association between ulcerative colitis and climatic variables.

We assume that temperature itself is not directly affecting the development of ulcerative colitis, but that temperature governs mechanisms which again are related to the likelihood for developing the disease. One such biologic mechanism is the number of species which is correlated to environmental temperature. According to the directed acyclic graphs literature, temperature is an instrumental variable for the effect of microbial species richness on ulcerative colitis because i) temperature affects species richness [35], ii) temperature affects the development of ulcerative colitis only through species richness and iii) there are no common cause of both temperature and disease. We can only assume the two latter conditions.

The number of species is not distributed homogeneously on Earth, but seems to follow some patterns [31], such as along a north–south axis. This pattern is characterized by steady decrease in the species richness from the tropics to northern or southern locations, and is called the latitudinal diversity gradient. This phenomenon is present for all taxes, but seems to be stronger for larger organisms than smaller organisms [36]. Other patterns also exist: the number of species is positively related to precipitation; positively related to area-size (islands); and negatively related to altitude. The causes of these patterns are not fully understood. Important for our study, environmental temperature is the strongest factor linked to biodiversity [31].

Our result suggests that the spatial variability in incidence rate is governed by the same factors as those related to species richness; and furthermore, species richness - not temperature - could be a strong force behind the likelihood for developing the disease. An important prerequisite is that a reduced microbial flora of harmless species may induce immunoregulatory reactions which are important for initiating autoimmune diseases like ulcerative colitis [37]. Consequently, reduced number of species to colonize the human gut will increase the likelihood for changes in the balance between gut flora and immunoregulation, which increases the likelihood for developing the disease [38].

Guernier et al. reported strong positive associations between diversity of pathogens known to affect human health and temperature, and negative associations for precipitation [39]. Among six different etiological groups, temperature was significantly related to bacteria, helminths, and viruses. There are no parallel investigations concerning commensals and temperature. However, a comparison of microbiota of children from urban Italy and rural Africa (Burkina Faso) revealed large differences in both composition and biodiversity [40]. In particular, the gut flora for Burkina Faso children had more Bacteriodetes than their Italian peers and less Firmicutes and Enterobacteriaceae [40]. This investigation demonstrated a higher microbial richness and biodiversity in African samples compared to European samples. The extended biodiversity and the increased amount of short-chain fatty acid producing bacteria found in Burkina Faso samples, probably due to the high-fiber diet, protected them from establishment of potentially pathogenic microbes. The authors suggested, in line with Rook et al. [30], that the microbial richness might be protective against, not only gastrointestinal pathogens, but also against autoimmune diseases like ulcerative colitis and Crohn’s disease.

We were not able to identify participants with Crohn’s disease in this study because there was no medication that could be used to identify participants with Crohn’s disease, such as 5-ASA medication prescribed to individuals suffering from ulcerative colitis. However, due to the high correlation between the incidence rates of ulcerative colitis and Crohn’s disease in Norwegian municipalities (0.42) [19] and the shared pathogenesis of the two diseases, it is likely that temperature is also a common risk factor for Crohn’s disease. Other autoimmune diseases such as multiple sclerosis [41], allergy [42], rheumatoid arthritis [43], systemic lupus erythematosus in US [44], but not in UK [45] have shown similar spatial patterns. No systematic studies exist for ankylosing spondylitis or diabetes type 1, but north–south trends or differences have been reported for both disorders [46,47]. Two studies have shown similar associations between temperature and incidence rates; multiples sclerosis [48] and asthma [49]. The mechanism we have introduced could also explain the geographic distribution observed for some of these diseases.

The hygiene hypothesis is debated mainly because no causal relationships between lifestyle factors associated with “hygiene” and incidence of disease have been revealed; however, animal models are promising (see [50] for review). Recent epidemiological studies have investigated hygiene-related factors and the risk of developing ulcerative colitis, but these potential factors were either not significant or confounded with other known risk factors [27,28]. In the present study, we investigated patterns of disease rates and temperature and its possible relation to biodiversity rather than single species or events. This highlights a difference between the hygiene hypothesis [26] and the “Old Friends” hypothesis [51]. The latter is more focused on absence or reduced numbers of commensal species rather than presence/absence of pathogens.

We cannot rule out the effect of sun-exposure and production of vitamin D, because sun-exposure is also related to latitude. Several studies have shown immunomodulatory effects of vitamin D, and vitamin D also prevents autoimmune responses (see [52] and references therein). Deficiency in vitamin D is well recognized among inflammatory bowel disease patients, but it is debated if this is a consequence of the disease (reverse causation) or directly causative [53]. In a meta-analysis studying polymorphisms of vitamin D receptor genes and risk of inflammatory bowel disease, Xue et al. found that males with specific genotypes were at higher risk for ulcerative colitis than healthy controls [54]. However, both mechanisms can be true, but we are at the present stage not able to estimate the relative contributions from vitamin D and the “Old friends” hypothesis.

The strength of this study is a relatively large number of cases sampled from a large area spanning the north–south axis and where other factors such as access to health care facilities and economic conditions are relatively equal. Furthermore, the present study comprised a relatively large range for the climatic variables. The span in temperature in this study corresponded to a difference between southern and northern Europe. We also included a set of potential contextual variables in our analyses; however, these variables should be included with care because area could both work as a confounder as well as a collider [55]. In the latter case (collider) the contextual variable would have introduced bias. The fact that more men than women suffered from the disease and that there were significantly less smokers among the affected persons reinforced the assumption that we had identified ulcerative colitis cases [56].

There were several weaknesses in our study. The first and most important was the use of self-reported medication and not diagnoses. Secondly, we have included prevalent and not incident cases. We have no information regarding when the subjects were diagnosed or their history of migration. In Norway, seven out of nine (78%) who moved during 1977–1998 did so within their economic region or within their municipality, the rest between counties [57]. These sources of information bias were most likely independent of temperature and can be classified as non-differential misclassification and will produce smaller effects than actually (attenuation). There are also uncertainties attached to environmental risk factors such as diet [58] and acute gastrointestinal infections [59].

## Conclusions

In this study we have investigated the geographic distribution of self-reported medication prescribed to patients with ulcerative colitis. We have found that the prevalence of the disease is associated to summer temperature. Temperature is a strong factor governing species richness, and our speculation is that as temperature decreases, the colonization of microbial species in the human gut also decreases, the likelihood for an imbalance in the gut flora increases, and the likelihood for developing the disease increases. This explanation of the spatial distribution of ulcerative colitis is in accordance with the extended hygiene hypothesis, and that the extended hygiene hypothesis could explain both temporal and spatial distribution of incidence rates of ulcerative colitis.

### Ethics

The study has been approved by the Regional Ethical Committee for South-eastern Norway and the CONOR steering group.

## Competing interests

The authors declare that they have none competing interests.

## Authors’ contributions

GA conceived the idea, performed the data-analysis and wrote the first draft of the manuscript. All authors contributed to the data-analysis, interpretation of the results, and writing of the manuscript. All authors read and approved the final manuscript.

## Pre-publication history

The pre-publication history for this paper can be accessed here:

http://www.biomedcentral.com/1471-2458/13/530/prepub
